# Immuno-Regulatory Function of Indoleamine 2,3 Dioxygenase through Modulation of Innate Immune Responses

**DOI:** 10.1371/journal.pone.0071044

**Published:** 2013-08-05

**Authors:** Malihe-Sadat Poormasjedi-Meibod, Raza B. Jalili, Azadeh Hosseini-Tabatabaei, Ryan Hartwell, Aziz Ghahary

**Affiliations:** Division of Plastic Surgery, Department of Surgery, University of British Columbia, Vancouver, British Columbia, Canada; University Medical Center Freiburg, Germany

## Abstract

Successful long-term treatment of type-1 diabetes mainly relies on replacement of β-cells via islet transplantation. Donor shortage is one of the main obstacles preventing transplantation from becoming the treatment of choice. Although animal organs could be an alternative source for transplantation, common immunosuppressive treatments demonstrate low efficacy in preventing xenorejection. Immunoprotective effects of indoleamine 2,3-dioxygenase (IDO) on T-cell mediated allorejection has been extensively studied. Our studies revealed that IDO expression by fibroblasts, induced apoptosis in T-cells while not affecting non-immune cell survival/function. Since macrophages play a pivotal role in xenograft rejection, herein we investigated the effect of IDO-induced tryptophan deficiency/kynurenine accumulation on macrophage function/survival. Moreover, we evaluated the local immunosuppressive effect of IDO on islet-xenograft protection. Our results indicated that IDO expression by bystander fibroblasts significantly reduced the viability of primary macrophages via apoptosis induction. Treatment of peritoneal macrophages by IDO-expressing fibroblast conditioned medium significantly reduced their proinflammatory activity through inhibition of iNOS expression. To determine whether IDO-induced tryptophan starvation or kynurenine accumulation is responsible for macrophage apoptosis and inhibition of their proinflammatory activity, Raw264.7 cell viability and proinflammatory responses were evaluated in tryptophan deficient medium or in the presence of kynurenine. Tryptophan deficiency, but not kynurenine accumulation, reduced Raw264.7 cell viability and suppressed their proinflammatory activity. Next a three-dimensional islet-xenograft was engineered by embedding rat islets within either control or IDO–expressing fibroblast-populated collagen matrix. Islets morphology and immune cell infiltration were then studied in the xenografts transplanted into the C57BL/6 mouse renal sub-capsular space. Local IDO significantly decreased the number of infiltrating macrophages (11±1.47 vs. 70.5±7.57 cells/HPF), T-cells (8.75±1.03 vs. 75.75±5.72 cells/HPF) and iNOS expression in IDO-expressing xenografts versus controls. Islet morphology remained intact in IDO-expressing grafts and islets were strongly stained for insulin/glucagon compared to control. These findings support the immunosuppressive role of IDO on macrophage-mediated xeno-rejection.

## Introduction

In spite of several attempts during last decades to overcome the xenotransplant hyper acute rejection mediated by pre-formed anti-αGal xeno-reactive antibodies, delayed xenograft rejection, mediated by progressive mononuclear cell infiltration, is still the main issue in preventing the widespread usage of animal organs for transplantation [1]. Histopathological studies [2], [3], [4] revealed a significant difference between mechanisms involved in cell mediated allogeneic and xenogeneic graft rejection. The main infiltrating cells in allograft rejection are TCR α/β positive cytotoxic T cells; while, xenografts are mainly infiltrated by NK cells and macrophages [3]. Further studies [5], [6] elucidated the interdependent roles of macrophages and T cells in xenograft rejection. Fox *et al.* study (2001) revealed that recognition of xenograft pathogen-associated molecular patterns (PAMPs) by innate immune receptors leads to macrophage infiltration into the graft [6]. The subsequent rapid and local innate immune response stimulates T cell infiltration. Infiltrated T cell subsequently activates macrophages to act as direct effector cells in xenograft rejection [5], [6]. Activated macrophages destruct the graft via secreting various proinflammatory mediators including TNF-α, reactive oxygen and nitrogen species, and complement factors [7]. The difference between immune responses involved in allo- and xenogeneic graft rejection could explain why the routine immunosuppressive strategies are ineffective in supporting xenograft against immune rejection.

Recent studies [8], [9] demonstrate that localized expression of immuno-regulatory factors, providing an immunoprivileged microenvironment, can be used as a feasible immunosuppressive strategy in post transplant patients. Indoleamine 2,3-dioxygenase (IDO), a cytosolic, heme containing enzyme, catalyses the first and rate-limiting step in metabolism of essential amino acid L-tryptophan to N-formylkynurenine [10]. The immuno-regulatory function of IDO was first described regarding its role in preventing T cell-mediated allogeneic fetus rejection in mice [11]. Further studies demonstrated the pivotal role of IDO in immuno-regulation of cancer [12], [13], inflammation and allergy, autoimmune disorders [14], and allotransplantation [8], [15]. Both local tryptophan deprivation and formation of toxic tryptophan catabolites contribute to immunosuppressive effects of IDO. The immuno-regulatory effects of IDO are mainly mediated through inhibition of T-cell proliferation [16], [17], prevention of memory T-cells formation [18] and induction of T-regulatory cells differentiation [19], [20], [21].

We have previously shown that IDO-expressing fibroblast co-culture with different subsets of primary human T cells leads to a significant reduction in T cell proliferation and survival [22]. Forouzandeh et al. studies [23] also revealed that selective activation of GCN2 kinase pathway in response to IDO induced tryptophan deficiency is responsible for T cell apoptosis. Further studies by our group [8], [24], [25] demonstrated that IDO expression by bystander fibroblasts can prevent cellular and humoral allo-immune responses against islet allotransplant without having any adverse effect on viability and functionality of the graft. Although these data support the feasibility of using local IDO expression as an alternative for systemic immunosuppressive treatments following allotransplantation, there are limited reports [26], [27] that have examined the use of IDO expression for graft protection following xenotransplantation. Li *et al.* study demonstrated a significant reduction in the CD3^+^ T lymphocytes infiltration into the IDO expressing skin xenograft compared to control [26]. Sandrin *et al.* (2008) also examined the effect of transgenic IDO expression on the outcome of porcine cell line (PIEC) transplantation into BALB/c mice. They showed that IDO expression by PIEC cell line significantly reduced the proliferation of PIEC primed BALB/c splenocytes, inhibited the production of Th1 and Th2 cytokines and markedly reduced the level of lymphocytes infiltration into the graft [27].Since none of these studies investigated the effect of local IDO expression on innate immune system mediated xenograft rejection, here we examined the immunomodulatory effect of IDO expression on innate immune responses involved in the cell mediated xenograft rejection. The results of this study provided evidence for the first time that IDO expression by bystander fibroblasts can inhibit macrophage and CD3^+^ cells infiltration into the islet xenogeneic graft and provide immuno-protection against innate immune responses. *In vitro* studies demonstrate that immuno-protective effects of IDO are mediated via tryptophan deficiency which induces apoptosis in macrophages and inhibit the proinflammatory activity of these cells.

## Materials and Methods

### Ethics Statement

This study was approved by the University of British Columbia Animal Care Committee. Care and maintenance of all animals used in this study were in accordance with the principles of laboratory animal care and the guidelines of the institutional Animal Policy and Welfare Committee at the University of British Columbia.

### Cell Culture

Dermal fibroblasts and primary macrophages were obtained from C57BL/6 (B6) mouse skin and peritoneal lavage, respectively. Raw264.7 cells, the murine macrophage cell line, and Jurkat cells, an immortalized line of T lymphocyte, were purchased from American Type Culture Collection (ATCC, Manassas, Virginia, United States).

Peritoneal macrophages, Raw264.7 cells, Jurkat cells and dermal fibroblasts were cultured in RPMI (Invitrogen Life Technologies, Carlsbad, CA) containing 10% heat-inactivated fetal bovine serum (FBS) and maintained in a 37°C humidified incubator containing 5% CO_2_.

### Cell Proliferation Assay

Raw264.7 cells, and dermal fibroblast, were plated in 96-well plates (50×10^3^ and 20×10^3^ cells/well, respectively) using RPMI containing 10% FBS. After overnight incubation, medium was changed and cells were incubated in RPMI, tryptophan-deficient medium (Trp-D, Gibco, USA), tryptophan-deficient medium supplemented with 50 µg/ml tryptophan (Trp-D+Trp) or RPMI supplemented with increasing concentrations of kynurenine (10, 25, 50, 100 and 200 µg/ml) for 24 hours. Subsequently, 3-(4, 5-dimethylthiazol-2-yl)-2, 5-diphenyltetrazolium bromide (MTT, Sigma-Aldrich) at 5 mg/ml was added to each well and incubated for 4 hours in the dark. The MTT/medium was carefully removed, and 100 µl dimethyl sulfoxide (DMSO, Sigma-Aldrich) was added, followed by incubation at 37°C for 10 minutes. The absorbance at 570 nm was measured using a BioTek Microplate spectrophotometer (Bio-Tek Instruments,Winooski, VT).

Jurkat cells were resuspended in different media as described above and plated in 96 well plates (50 ×10^3^ cells/well). After 24 hours of incubation MTT was added to the wells and incubated for 4 hours in the dark. Cells were spun down (1500 RPM, 5 min, RT), 50 µl of the medium was removed and DMSO was added to the wells. The absorbance was measured at 570 nm.

### Live/dead, Viability/cytotoxicity Assay

The cellular viability of Raw264.7 cells was determined by flowcytometry using the Live/Dead, Viability/Cytotoxicity assay Kit for mammalian cells (Invitrogen) which provides a two-color fluorescence cell viability assay. Raw264.7 cells were plated in 12-well plates (1×10^6^ cells/well) and cultured as described above. Cells were incubated with Calcein AM (2 mM)/ethidium homodimer (4 mM) (Molecular Probes, Canada) solution for 30 minutes in dark, cells were harvested and flow cytometry was done. After gating on the basis of forward versus side scatter to exclude cell debris and clumps, percentage of dead cells plus cells undergoing apoptosis with damaged cellular membrane (PE positive) was quantitatively evaluated based on the protocol suggested by the manufacturer (Molecular Probes, Invitrogen, Mississauga Canada).

### Preparation of Cell Lysates

Raw264.7 cells, dermal fibroblasts (2×10^6^ cells/10 cm^2^ plates), and Jurkat cells (2×10^6^ cells/75 cm^2^ flask) were cultured and treated as described above. Cells were harvested and centrifuged (1500 RPM, 4°C, 5 min). Cell pellets were then resuspended in 100 µl of cell lysis buffer (50 mM Tris-HCl, pH 7.4, 10 mM EDTA, 5 mM EGTA, 0.5% NP40, 1% Triton X-100, and protease inhibitor cocktail (Sigma-Aldrich)). After cell lysate centrifugation (14000 RPM, 4°C, 5 min), protein concentration of supernatant was quantified using BCA protein assay kit (Pierce, Rockfield, IL).

### Western Blot Analysis

Proteins of the whole-cell lysates (40 µg) were separated by running the sample on 10% SDS- polyacrylamide gel and then transferred to PVDF membrane (Millipore, Bedford, MA). The membranes were blocked and blots were probed for CCAAT/enhancer-binding protein homologous protein (CHOP), poly (ADP-ribose) polymerase (PARP, full length and cleaved protein), and caspase-3 (full length and cleaved protein) using rabbit-anti CHOP-10 Ab (1∶250 dilution, #2895 Cell signalling), rabbit-anti PARP Ab (1∶1000 dilution, #9542 Cell signalling), and rabbit-anti caspase-3 Ab (1∶1000 dilution, #9662 Cell signalling), respectively. Horseradish peroxidase (HRP)-conjugated goat anti-rabbit Ab (1∶3000 dilution, Bio-Rad) was used as the secondary antibody. Enhanced chemiluminescence detection system (ECL; Amersham Biosciences, UK) was used in all blots to detect the related secondary antibody. Beta-actin (β-actin) was used as the protein loading control.

### Measurement of the Inducible Nitric Oxide Synthase (iNOS) Expression by Real Time Quantitative-PCR

Effect of tryptophan deficiency and kynurenine enrichment on macrophage proinflammatory activity was determined by evaluating iNOS expression and nitric oxide (NO) production in response to lipopolysaccharide (LPS) and interferon-gamma (IFN-γ) stimulation. Raw264.7 cells (2×10^6^ cells) were plated using fresh medium. After overnight incubation, medium was changed and cells were incubated in RPMI, tryptophan-deficient medium (Trp-D), tryptophan-deficient medium supplemented with 50 µg/ml tryptophan (Trp-D+Trp) or RPMI supplemented with increasing concentrations of kynurenine (10, 25, and 50 µg/ml) for an hour. Subsequently cells were stimulated with 10 ng/mL LPS and 10 U/mL IFN-γ. After 12 hours of incubation, cells were collected and total RNA was isolated and purified using RNeasy (Qiagen, Valencia, CA) as recommended by the manufacturer. The purity and concentration of the extracted RNA were checked by measuring the absorbance ratio at 260/280 nm. Following DNase treatment, total RNA (5 µg) was reverse transcribed into cDNA using a Superscript II First Strand cDNA Synthesis kit (Invitrogen). Q-PCR was performed on Applied Biosystems 7500 PCR machine using the following PCR cycling conditions: 95°C for 5 min, 40 cycles at 95°C for 15 Sec and 60°C for 1 min. iNOS mRNA levels were measured in triplicates and Glyceraldehyde-3-phosphate dehydrogenase (GAPDH) was used as the reference household gene to normalize the amount of mRNA in the cultures. The gene expression fold change, relative to the control was calculated by 2^− ΔΔCT^. The following primers were used for Q-PCR reactions: iNOS, 5′-CCCTCCAGTGTCGGGAGCA-3′ and 5′ TGCTTGTCACCACCAGCAGT-3′ and GAPDH, 5′-TCACCACCATGGAGAAGGC-3′and 5′GCTAAGCAGTTGGTGGTGCA-3′ (Invitrogen).

### Griess Assay for Measurement of NO Production by Activated Macrophages

To study the NO production in response to LPS+IFN-γ stimulation, Raw264.7 cells were cultured and stimulated as described in the previous section for 24 hours. The NO concentration in the cell culture supernatants was quantified photometrically using the Griess assay [28]. Each experiment was performed four times in triplicate and absorbance was compared with a standard curve plotted against different concentrations of sodium nitrite.

### Peritoneal Macrophage-fibroblast Co-culture Systems

Dermal fibroblasts were infected with a recombinant adenoviral vector carrying human IDO cDNA. IDO expression and activity was evaluated in the transfected fibroblasts using Western blotting and kynurenine assay as described previously [23], [29]. Control fibroblasts were left untreated.

In order to confirm the data obtained from cell line studies and evaluate the effect of IDO expression by bystander fibroblasts on the viability of peritoneal macrophages, a macrophage/fibroblast co-culture system was used. In this setting, fibroblasts and macrophages were grown in the upper and lower chambers of the system, respectively. For co-culture experiments, 12 and 30 mm Millicell-CM (Millipore) culture plate inserts (0.4 µm pore size) were used. Either control or IDO-expressing fibroblasts were seeded on the inserts (0.1×10^6^ and 0.3 ×10^6^ fibroblasts for 12 and 30 mm inserts, respectively) and cultured in RPMI with 10% FBS overnight. In a separate experiment 0.5×10^6 ^or 2×10^6^ cells obtained from peritoneum lavage were seeded in each well of a 12- or 6-well culture plates, respectively. The cells were incubated overnight in RPMI with 10% FBS allowing the macrophages to adhere; then, the plates were washed twice with warm RPMI to remove non-adherent cells. The co-culture system was then assembled, with the upper chamber holding the insert with fibroblasts and the lower chamber containing the peritoneal macrophages. The control was primary macrophages cultured in RPMI. Following 3 days of incubation, inserts were removed and primary macrophages, cultured in the lower chamber, were subjected to MTT assay and Western blotting to evaluate the cell viability and examine the presence of cleaved caspase-3 and CHOP in the cell lysate.

The inhibitory effect of IDO-induced tryptophan deprivation on the proinflammatory activity of primary macrophages was determined by evaluating iNOS expression in response to LPS+IFN-γ stimulation. Cells obtained from peritoneal lavage were seeded in 6-well plates (2×10^6 ^cell/well). Wells were washed following overnight incubation and peritoneal macrophages were treated with either normal or IDO-expressing fibroblast conditioned medium, having high levels of kynurenine and low levels of tryptophan, for an hour. Subsequently, cells were stimulated with LPS+IFN-γ and iNOS expression was evaluated in primary macrophages after 12 hours of incubation using Q-PCR as described above.

### Primary Macrophage Treatment with IFN-γ and IDO Expression Evaluation

Peritoneal macrophages, cultured in RPMI, tryptophan deficient medium (T), or tryptophan deficient medium supplemented with 50 µg/ml tryptophan (T+T), were treated with 1000 U/ml of IFN-γ for either 24 hours (mRNA analysis) or 48 hours (protein analysis). IDO mRNA and protein expression was evaluated in the cell pellet using RT-PCR and Western blotting, respectively. The following primers were used for RT-PCR: IDO, 5′-GGCACACGCTATGGAAAACT-3′, and 5′-CGGACATCTCCATGACCTTT-3′, and β-actin, 5′-GTGGGCCGCCCTAGGCACCAA-3′, and 5′-CTCTTTGATGTCACGCACGATTTC-3′. Polyclonal rabbit anti-human IDO antibody (1∶1000; Washington Biotechnology Inc, Baltimore, MD, USA) was used to detect IDO in Western blotting.

### Rat Islet Isolation, Preparation and Transplantation of Three-dimensional Islet-fibroblast Composite Grafts

Rat islets were obtained from male Long Evans rats (The Jackson Laboratories, Bar Harbor, ME) as previously described [25] with some modifications. In brief, 10 ml of Hanks’ balanced salt solution (HBSS; Life Technologies, Gaithersburg, MD) containing 0.75 mg/ml of collagenase (Type V; Sigma-Aldrich, St. Louis, MO) was injected into the rat pancreases through the pancreatic duct. The distended pancreases were then removed and incubated at 37°C for 10 min. Collagenase enzymatic activity was stopped by adding HBSS supplemented with 0.25% BSA and tissue suspension was filtered through a 400 µm screen to remove undigested particles. The filtered digested tissue was passed through a 100 µm nylon cell strainer (Fisherbrand, Fisher Scientific International Inc., Pittsburgh, PA), and the screen was rinsed extensively with HBSS supplemented with 0.25% BSA. Islets were handpicked and cultured in HAM’s F10 medium (Sigma-Aldrich) supplemented with 12 mmol/L HEPES, 2 mmol/L L-glutamine, 10% heat-inactivated fetal calf serum (FCS), 100 U/ml penicillin, and 100 mg/ml streptomycin in 95% air, 5% CO_2_ at 37°C overnight. IDO-expressing or control fibroblasts were used to prepare fibroblast-populated collagen gel (FPCG) matrices as described elsewhere [30]. Rat islets (300 islets per graft) were added to FPCG before solidification. Islet-fibroblast composite grafts were then transplanted under the left kidney capsule of B6 mice (n = 4 for each case or control group).

### Tissue Processing and Immunohistochemistry

Graft-bearing kidneys were harvested 10 days after transplantation, fixed in 10% buffered formalin solution, and embedded in paraffin. Hematoxylin-eosin (H-E) staining and double immuno-fluorescence staining for either insulin and glucagon, insulin and CD3, insulin and F4/80 or iNOS and F4/80 was done for 5 µm thick sections of graft area. Sections were rehydrated, and nonspecific binding was blocked. Sections were then incubated overnight at 4°C with appropriate primary antibodies, including guinea pig anti-insulin antibody (1∶500 dilution; Dako Laboratories, Mississauga, Ontario, Canada), rabbit anti-glucagon (1∶750, Dako), rabbit anti-CD3 antibody (1∶50 dilution; Abcam, Cambridge, MA), rat anti-mouse F4/80 (1∶50 Serotec, Oxford, England) and rabbit anti-iNOS (1∶100 Pierce antibodies, thermo Scientific). Sections were then washed and incubated with relevant secondary antibodies, including fluorescein isothiocyanate (FITC) goat anti-guinea pig IgG (Abcam), and Rhodamine goat anti-rabbit IgG (Chemicon International, Temecula, CA), FITC goat anti-Rabbit IgG (Chemicon International, Temecula, CA), and Rhodamine goat anti-rat IgG (Jackson ImmunoResearch, West Grove, PA) for 45 minutes. Finally, the slides were dehydrated and mounted in Permount (Fisherbrand). T cell and macrophage infiltration into the transplanted xenografts was quantified by manually counting the number of infiltrating CD3^+^ T cells and F4/80^+^ macrophages in four randomly selected high power fields (HPF) per tissue section. The average number of these four HPFs was reported as the cell number per HPF.

### Statistical Analysis

Data were expressed as mean±SEM of three or more independent observations. Statistical significance was calculated using a two-tailed unpaired Student t-test or a one-way analysis of variance with post hoc test in case of multiple comparisons. P-values<0.05 were considered statistically significant in this study.

## Results

### Tryptophan Deficiency, Rather than Kynurenine Accumulation Reduced the Proliferation and Viability of Raw264.7 Cells

**Figure 1:**
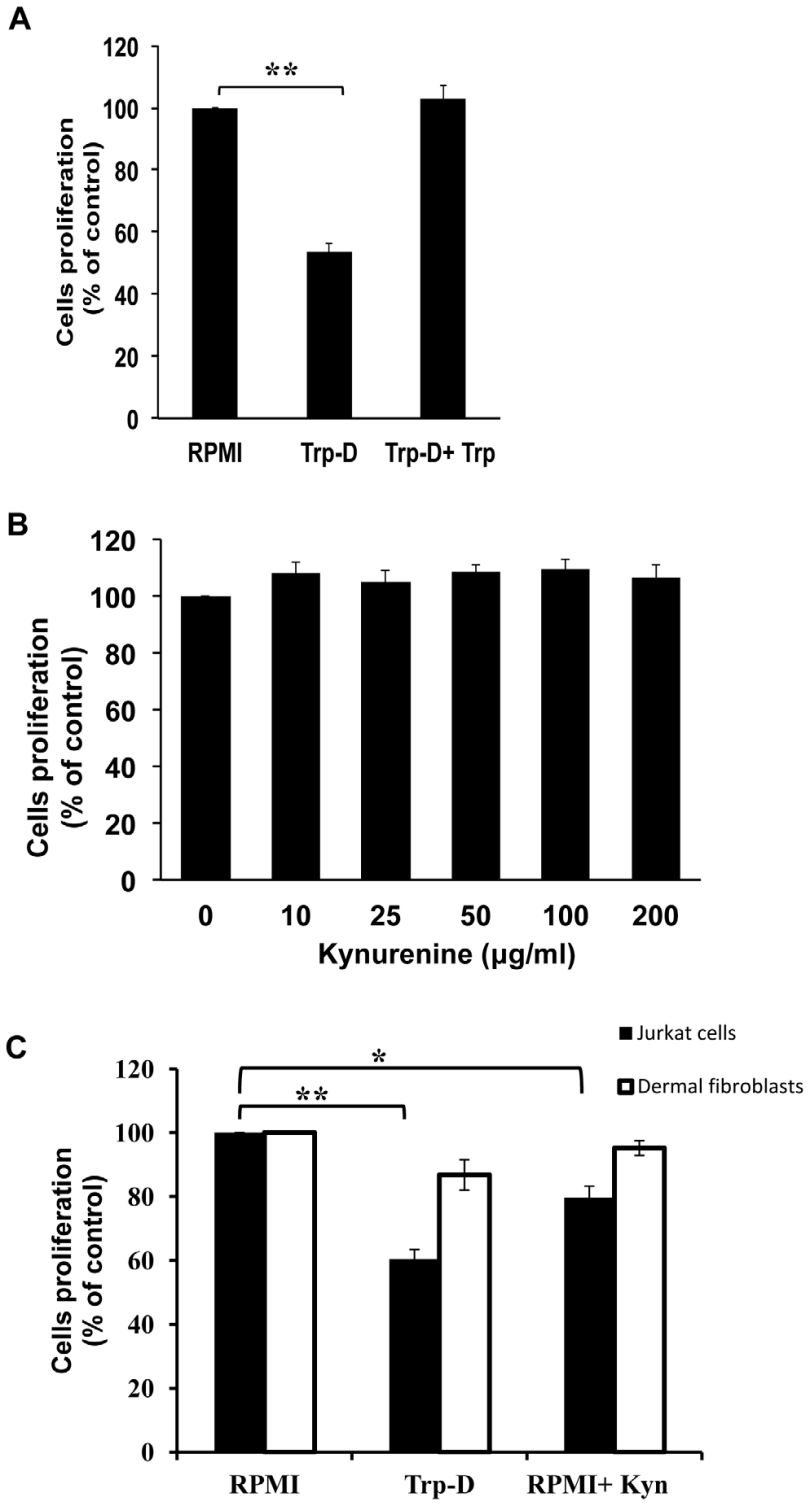
Effect of tryptophan deficiency and kynurenine accumulation on Raw264.7 cell, fibroblast and Jurkat cell proliferation. (A) Determination of the tryptophan deficiency effect on Raw264.7 cells proliferation and viability. Raw264.7cells were either cultured in RPMI, tryptophan-deficient medium (Trp-D) or tryptophan-deficient medium supplemented with 50 µg/ml tryptophan (Trp-D+Trp). MMT assay was done after 24 hours of treatment. (B) Evaluation of the kynurenine accumulation effect on Raw264.7 cells proliferation using MTT assay following 24 hours of treatment. (C) Evaluation of the effect of the tryptophan deficiency and high levels of kynurenine on Jurkat cells (solid bars) and dermal fibroblast (open bars) proliferation and viability using MTT assay after 24 hours of treatment. Data is mean±SEM of four independent experiments. (*P-value<0.05 and **P-value<0.01, n = 4).

In order to determine the effect of tryptophan deficiency on macrophage proliferation, an MTT assay was done on Raw264.7 cells cultured in either RPMI, tryptophan-deficient medium (Trp-D) or tryptophan-deficient medium supplemented with excessive amount of tryptophan (Trp-D+Trp). As shown in Figure 1A, a marked reduction in cellular proliferation was observed in Raw264.7 cells cultured in Trp-D as compared with the cells cultured in RPMI (53.67±5.46%, ** P-value<0.01, n = 4) after 24 hours. In order to confirm the inhibitory effect of tryptophan deficiency on Raw264.7 cell proliferation, excessive amount of tryptophan (50 µg/ml) was added to the tryptophan-deficient medium. Culturing Raw264.7 cells in Trp-D+Trp medium resulted in restoration of cell proliferation (Fig. 1A). MTT assay was also performed on Raw264.7 cells cultured in RPMI with increasing concentrations of kynurenine (10, 25, 50, 100 and 200 µg/ml) and the result shown in Figure 1B revealed no marked inhibitory effect on Raw264.7 cell proliferation (P-value = 0.478, n = 4). Our previous studies [22], [23] demonstrated the selective susceptibility of adaptive immune cells (T cells) to IDO induced tryptophan deficiency and high levels of kynurenine in comparison to dermal fibroblasts. As shown in Figure 1C (solid bars) Jurkat cell proliferation was significantly reduced in response to either tryptophan deficiency (60.37±6.10%, ** P-value<0.01, n = 4) or addition of 50 µg/ml of kynurenine (79.67±7.17%, * P-value<0.05, n = 4) as compared to that of cells in RPMI. Additionally, as shown in figure 1C (open bars), presence of high levels of kynurenine (50 µ/ml) did not have any noticeable adverse effect on dermal fibroblasts proliferation (95.21±4.57, P-value = 0.127, n = 4). Although tryptophan deficiency reduced fibroblasts proliferation, this reduction was not statistically significant (86.77±9.46, P-value = 0.068, n = 4).

**Figure 2:**
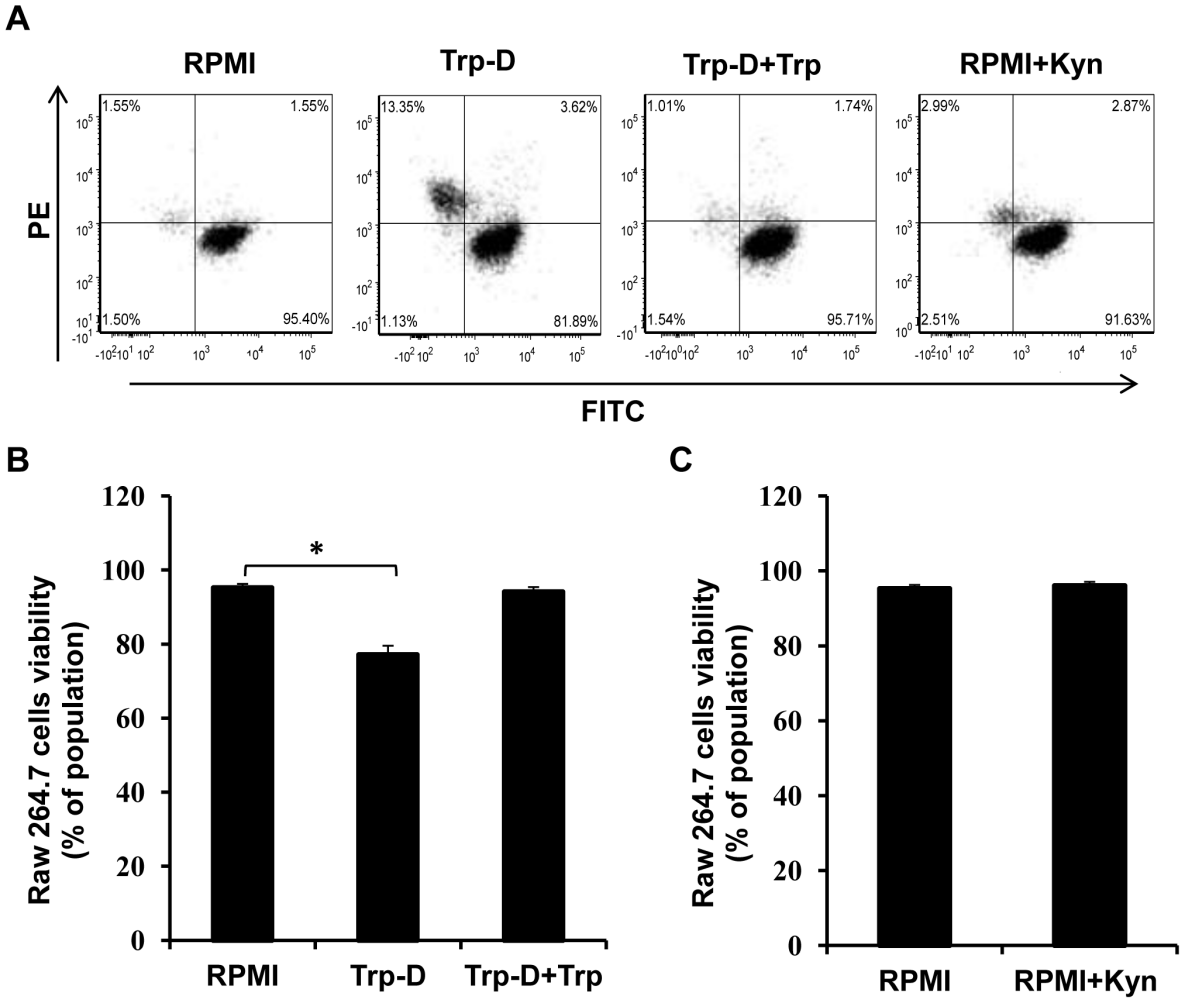
The effect of tryptophan deficiency and high levels of kynurenine on Raw264.7 cell survival. (A) Determination of cellular viability by FACs analysis using live/dead, viability/cytotoxicity assay kit. Raw264.7cells were either cultured in RPMI, tryptophan-deficient medium (Trp-D), tryptophan-deficient medium supplemented with 50 µg/ml tryptophan (Trp-D+Trp) or RPMI supplemented with 50 µg/ml kynurenine (RPMI+Kyn). The viability of Raw264.7 cells was determined by FACS analysis. (B) Raw264.7 cell survival is represented by the percentage of viable cells in the total cell population. Data is mean±SEM of three independent experiments (*P-value<0.05, n = 3).

The effect of tryptophan deficiency and high levels of kynurenine on Raw264.7 cell survival was evaluated by flowcytometry using a viability/cytotoxicity assay. In this assay clacein AM is converted to a green fluorescent compound by active intracellular esterase in live cells with intact membranes (FITC positive cells); while, ethidium homodimer (EthD-1), a membrane impermeable, red fluorescent nucleic acid dye, stains dead cells and those undergo apoptosis (PE positive cells). As shown in Figure 2A, the percentage of dead cells in addition to cells with damaged membrane undergoing apoptosis increased up to 5.46 times in response to tryptophan deficiency (16.97% PE positive cells in Trp-D medium in comparison to 3.11% in RPMI). The macrophage survival was restored by addition of excessive amount of tryptophan (50 µg/ml) to the Trp-D medium which confirms the Raw264.7 cells susceptibility to tryptophan deficiency (Fig. 2A, Trp-D+Trp). It was also shown that exposure to kynurenine (50 µg/ml) did not significantly increase the number of dead cells (Fig. 2A, RPMI+Kyn), indicating that kynurenine is non-toxic to Raw264.7 cells at this dose. Viability of Raw264.7 cells was also evaluated across all treatments and compared against RPMI as a control. As shown in figure 2B, tryptophan deficiency (Trp-D) significantly reduced the percentage of live cells (77.16±4.15%, * P-value<0.05, n = 3) in comparison to those cultured in RPMI. It was also shown that the survival of Raw264.7 cells cultured in Trp-D medium supplemented with excessive amounts of tryptophan is comparable to those cells cultured in RPMI (Fig. 2B, Trp-D+Trp). Furthermore, it was shown that high levels of kynurenine (50 µg/ml) resulted in a negligible cytotoxic effect on Raw264.7 cells, with less than 1% reduction in cell survival (Fig. 2C).

### Tryptophan Deficiency Induces Macrophage Apoptosis

**Figure 3:**
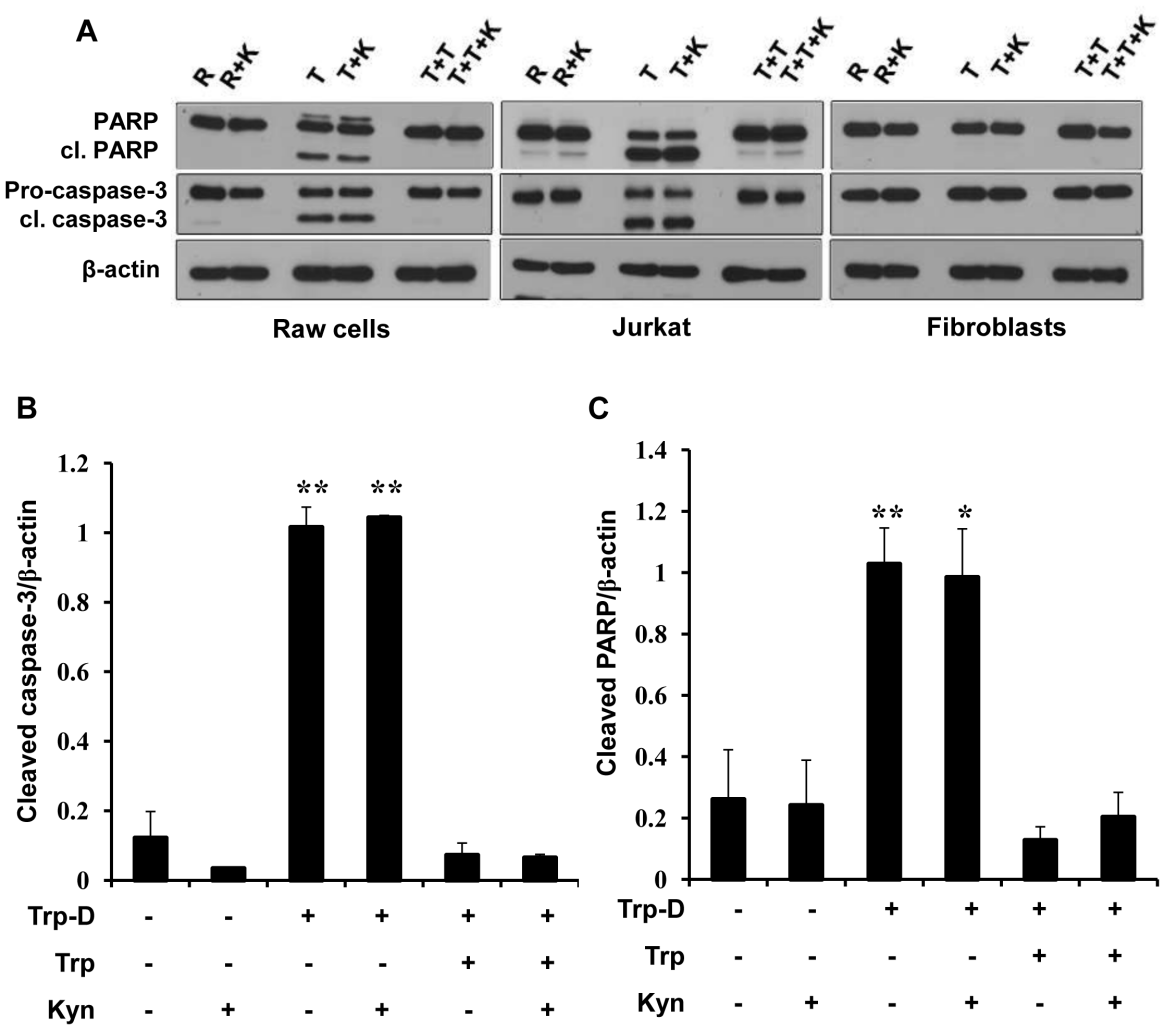
Apoptosis induction in Raw264.7 cells in response to tryptophan deficiency. (A) Raw264.7 cells were cultured in RPMI (R), tryptophan-deficient medium (T), tryptophan-deficient medium supplemented with 50 µg/ml tryptophan (T+T) in the presence or absence of 50 µg/ml kynurenine (K) for 24 hours. Presence of PARP, cleaved PARP (cl. PARP), Pro-caspase-3 and cleaved caspase-3 (cl. caspase-3) were analyzed by Western blotting. β-actin was used as the protein loading control. Jurkat cells and dermal fibroblasts were cultured in the same conditions. Results are representative of four independent experiments. (B) The ratio of cleaved-caspase-3/β-actin expression in Raw264.7 cells. (C) The ratio of cleaved PARP/β-actin expression in Raw264.7 cells. Data is mean±SEM of four independent experiments (*P-value<0.05 and **P-value<0.01, n = 4).

To determine the mechanisms involved in Raw264.7 cell death, the presence of cleaved caspase-3 and cleaved PARP, as two indicators for apoptosis were examined. To achieve this, caspase-3 activation and PARP cleavage were analyzed on cell lysates from cells cultured in RPMI (R), tryptophan-deficient medium (T), tryptophan-deficient medium supplemented with 50 µg/ml tryptophan (T+T) in the presence or absence of 50 µg/ml kynurenine (K). In the western blots probed with anti PARP and anti caspase-3 antibodies, the upper bands are the full length PARP protein (116 KD) and pro-caspase-3 (35 KD), respectively. The lower bands are the cleaved PARP (cl. PARP, 89 KD), signature fragment produced by caspase cleavage during apoptosis, and cleaved-caspase-3 (cl. caspase-3, 17 KD). As shown in figure 3A, while no PARP or caspase-3 cleavage is detectable in Raw264.7 cell cultured in RPMI (lane R), a marked increase in the levels of cleaved-PARP (cl. PARP) and cleaved-caspase-3 (cl. caspase-3) was observed in Raw264.7 cell cultured in tryptophan-deficient medium (lane T) or tryptophan-deficient medium supplemented with kynurenine (lane T+K) relative to the cells cultured in RPMI (lane R). It was also shown that cleavage of pro-caspase-3 and PARP was inhibited by addition of excessive amounts of tryptophan to the tryptophan-deficient medium (lane T+T). ‘Addition of kynurenine to RPMI or Trp-D+Trp medium did not have any noticeable effect on the detectable levels of cleaved caspase-3 and cleaved PARP (lane R+K and lane T+T+K, respectively) compared to cells cultured in RPMI (lane R). Jurkat cells and dermal fibroblasts were cultured in the same conditions as positive and negative controls for apoptosis induction in response to tryptophan deficiency and kynurenine accumulation, respectively. As it was shown previously by our group [22], [23], tryptophan deficiency induced a noticeable increase in PARP and pro-caspase-3 cleavage in Jurkat cells. This apoptotic response was inhibited by addition of excessive amounts of tryptophan to the Trp-D medium (Fig. 3A, middle panel). Dermal fibroblasts did not demonstrate detectable levels of apoptotic indicators, activated caspase-3 and cleaved PARP, in response to tryptophan starvation or high levels of kynurenine (Fig. 3A, right panel). For control of protein loading and quantitative analysis, all corresponding blots for Raw264.7 cells were re-probed for beta-actin (β-actin). The signals were then quantified by densitometry and the ratio of cleaved-caspase-3/β-actin or cleaved-PARP/β-actin was determined as shown in figure 3B and figure 3C, respectively. The finding clearly indicates a significant increase in the cleaved caspase-3 (Fig. 3B, ** P-value<0.01, n = 4) and cleaved PARP (Fig. 3C, * P-value<0.05, ** P-value<0.01, n = 4) level in Raw264.7 cells cultured in tryptophan-deficient medium, in the presence or absence of kynurenine, relative to cells cultured in RPMI. It was also shown that addition of excessive amounts of tryptophan (50 µg/ml) to the Trp-D medium reduced the detected levels of cleaved caspase-3 (Fig. 3B) and cleaved PARP (Fig. 3C) to the levels comparable to the cells cultured in RPMI. The addition of high levels of kynurenine (50 µg/ml) to RPMI or Trp-D+Trp medium did not increase the cleaved caspase-3 (Fig. 3B) or cleaved PARP (Fig. 3C) level in Raw264.7 cells.

### The Apoptotic Effect of Tryptophan Deficiency in Raw264.7 Cells is Caused by Activation of General Control Nonrepressed 2 (GCN2 Kinase) Pathway

**Figure 4:**
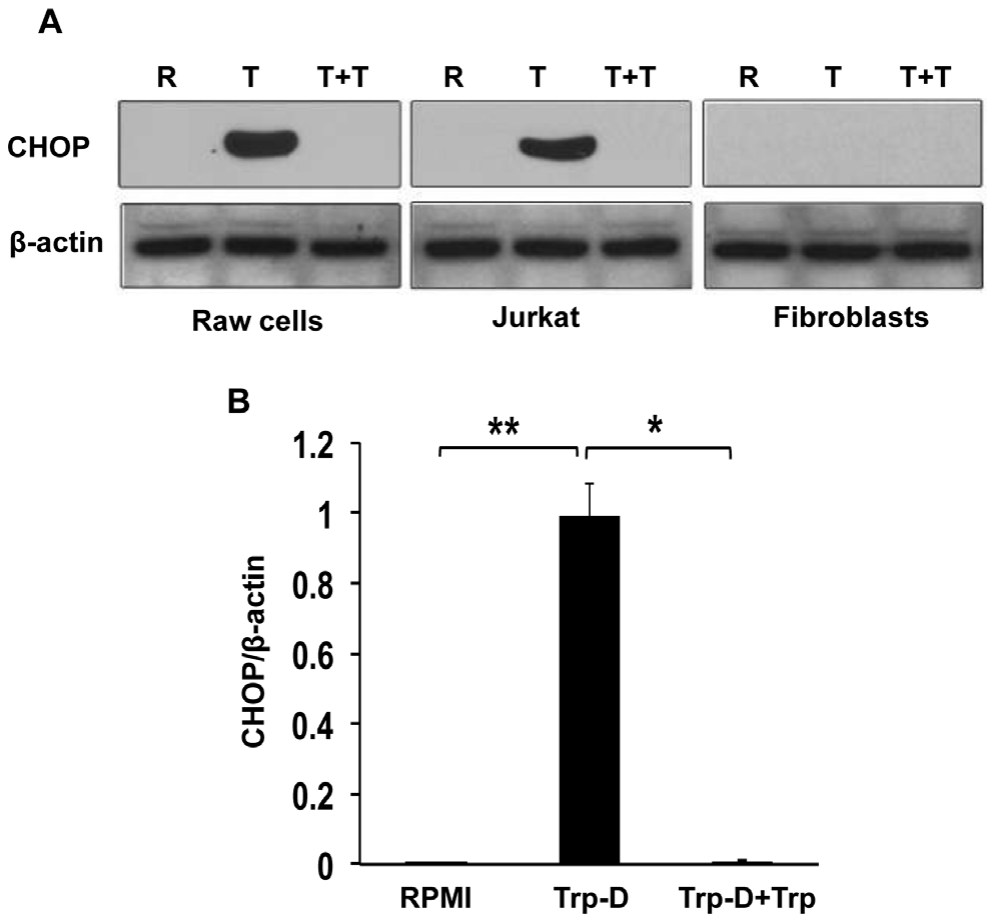
Specific activation of GCN2 kinase pathway mediates selective suppressive effects of tryptophan deficiency on Raw264.7cells. (A) Raw264.7 cells were cultured in RPMI (R), tryptophan-deficient medium (T) or tryptophan-deficient medium supplemented with 50 µg/ml tryptophan (T+T) for 24 hours. CHOP expression was analyzed by Western blotting. β-actin was used as the protein loading control. Jurkat cells and dermal fibroblasts were cultured in the same conditions. Results are representative of five independent experiments. (B) CHOP/β-actin expression ratio was calculated in Raw264.7 cells. Data is mean±SEM of five independent experiments (*P-value<0.05 and **P-value<0.01, n = 5).

It was previously shown [23] that the selective sensitivity of immune cells (T cells) to the IDO-induced low-tryptophan, high-kynurenine environment compared to fibroblasts was governed by their difference in GCN2 kinase pathway activation. GCN2 kinase pathway activation in response to tryptophan starvation results in proliferative arrest, anergy induction and apoptosis in T cells [22], [31]. In order to determine the effect of tryptophan deficiency on GCN2 kinase pathway activation in Raw264.7 cells, the expression of CHOP protein, a well-accepted marker for GCN2 activation, was evaluated in cells cultured in RPMI, tryptophan-deficient medium or tryptophan deficient medium supplemented with excessive amounts of tryptophan. As shown in figure 4A, left panel, CHOP expression was increased in response to tryptophan deficiency (lane T) in Raw264.7 cells relative to cells cultured in RPMI (lane R). It was also shown that CHOP expression was inhibited by addition of tryptophan to the Trp-D medium (lane T+T). As it was shown previously by our group [22], [23], tryptophan starvation (lane T) induced a marked increase in CHOP expression in Jurkat cells compared with cells cultured in RPMI (Fig. 4A, middle panel). This increase in CHOP expression by Jurkat cells was suppressed by the addition of excessive amounts of tryptophan to the Trp-D medium (Fig. 4A, middle panel, lane T+T). Dermal fibroblast did not express detectable levels of CHOP in response to tryptophan starvation (lane T), indicating the selective activation of GCN2 kinase pathway in immune cells (Fig. 4A, right panel). For quantitative analysis, the intensity of signals was determined by densitometry, and the mean±SEM of the CHOP expression/β-actin was plotted for Raw264.7 cells cultured in different media. A significant increase in CHOP expression was observed in response to tryptophan starvation (Trp-D), relative to those cells cultured in either RPMI (Fig. 4B, **P-value<0.01, n = 5) or tryptophan-deficient medium supplemented with tryptophan (Trp-D+Trp, Fig. 4B, *P-value<0.05, n = 5).

### Tryptophan Deficiency Inhibited iNOS Expression and NO Production by Stimulated Raw264.7 Cells

**Figure 5:**
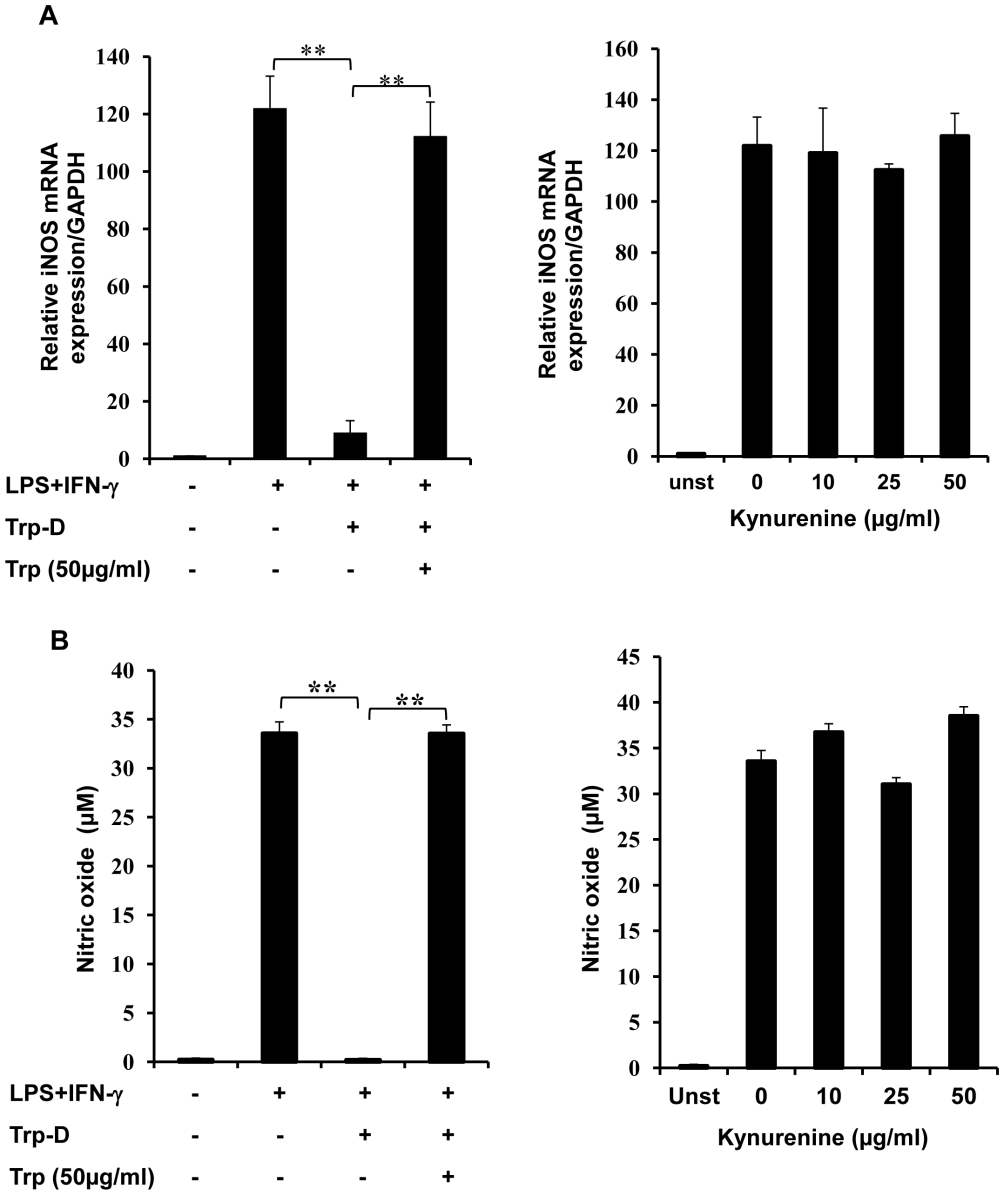
Inhibition of iNOS expression and NO production by tryptophan deficiency in IFN-γ+LPS stimulated RAW264.7 cells. (A) Q-PCR analysis of iNOS mRNA expression. Raw264.7 cells were pre-incubated in RPMI, tryptophan-deficient medium (Trp-D), tryptophan-deficient medium supplemented with 50 µg/ml tryptophan or RPMI supplemented with increasing concentrations of kynurenine (10, 25, and 50 µg/ml) for an hour. Cells were then stimulated with IFN-γ+LPS for 12 hours. Cells were collected, and iNOS expression was determined by Q-PCR after RNA extraction and cDNA synthesis. GAPDH was used as the reference gene. The relative expression of iNOS mRNA is evaluated by Q-PCR in Raw264.7 cells cultured and treated in different conditions. The iNOS expression was normalized to the GAPDH expression level (B) Raw264.7 cells were cultured and stimulated for 24 hours as described above. The concentration of released nitric oxide was evaluated in the cell supernatant by the Griess method. Data is mean±SEM of four independent experiments (**P-value<0.01, n = 4).

It is widely accepted that immunomodulatory effect of IDO on adaptive immunity is mediated not only by inhibition of T cell proliferation but also by anergy induction in effector T cells [31] and impairment of T cell allogeneic responses [32]. The presented MTT and viability/cytotoxicity assay results demonstrate that tryptophan starvation markedly reduced Raw264.7 cell proliferation and survival. In order to determine the effect of tryptophan starvation and kynurenine accumulation on the proinflammatory activity of the remaining live cells, iNOS expression and NO production were evaluated in stimulated Raw264.7 cells. Cells, cultured in RPMI, tryptophan-deficient medium (Trp-D) or tryptophan-deficient medium supplemented with 50 µg/ml tryptophan, were stimulated with LPS (10 ng/ml) and IFN-γ (10 U/ml). Then iNOS mRNA expression was evaluated by Q-PCR in which iNOS gene expression was normalized to GAPDH mRNA expression. As expected unstimulated Raw264.7 cells cultured in RPMI did not express a detectable level of iNOS, whereas cells that were exposed to LPS and IFN-γ demonstrated a marked increase in iNOS expression (Fig. 5A, left panel). Moreover, stimulated Raw264.7 cells cultured in tryptophan-deficient medium demonstrated a significant reduction in iNOS expression (fig. 5A, Left panel, ** P-value<0.01, n = 4) compared to cells cultured in RPMI. To verify the suppressive effect of tryptophan deprivation on Raw264.7 cell iNOS expression in responses to LPS and IFN-γ stimulation, these cells were cultured and stimulated in Trp-D medium supplemented with tryptophan (50 µg/ml). In contrast, stimulated Raw264.7 cells cultured in the presence of tryptophan expressed significantly higher amounts of iNOS relative to those cultured in the absence of tryptophan (Fig. 5A, Left panel, ** P-value<0.01, n = 4), which is comparable to stimulated cells cultured in RPMI. In order to determine the effect of kynurenine accumulation on macrophage pro-inflammatory responses, iNOS expression was evaluated in stimulated Raw264.7 cells cultured in RPMI with increasing concentration of kynurenine (10, 25 and 50 µg/ml). Stimulated cells cultured in RPMI or RPMI with increasing concentration of kynurenine expressed comparable levels of iNOS in response to stimulation (Fig. 5A, right panel).

The Griess assay was used to determine the iNOS enzymatic activity and the concentration of released NO into the medium after 24 hours of stimulation. As expected, unstimulated Raw264.7 cells did not release detectable levels of NO, whereas stimulated cells cultured in RPMI or Trp-D medium supplemented with excessive amounts of tryptophan released marked amounts of NO (33.55±2.6464 and 33.53±2.01 µM, respectively). It was also shown that stimulated Raw264.7 cells cultured in Trp-D medium demonstrated a noticeable reduction in NO production compared to stimulated cells cultured in RPMI or Trp-D medium supplemented with excessive amounts of tryptophan (Fig. 5B, left panel, ** P-value<0.01, n = 4). Moreover, it was shown that increasing concentrations of kynurenine (10, 25 and 50 µg/ml) did not have any inhibitory effect on NO production by stimulated cells cultured in RPMI (Fig. 5B, right panel).

### Apoptosis Induction in Peritoneal Macrophages Co-cultured with IDO Expressing Fibroblasts

Before evaluating the immunosuppressive effect of IDO on primary macrophages, IDO expression/functionality was validated in fibroblasts infected with an adenoviral vector carrying human IDO gene. The results of Western blotting demonstrates a strong IDO protein expression in fibroblasts infected with Ad-IDO (Fig. S1A and B, IDO-Fib, ** P-value<0.01, n = 3) as compared with normal fibroblasts (Fib). This finding was confirmed by detecting high levels of kynurenine in IDO-expressing fibroblast conditioned medium (Fig. S1C, ** P-value<0.01, n = 3).

**Figure 6:**
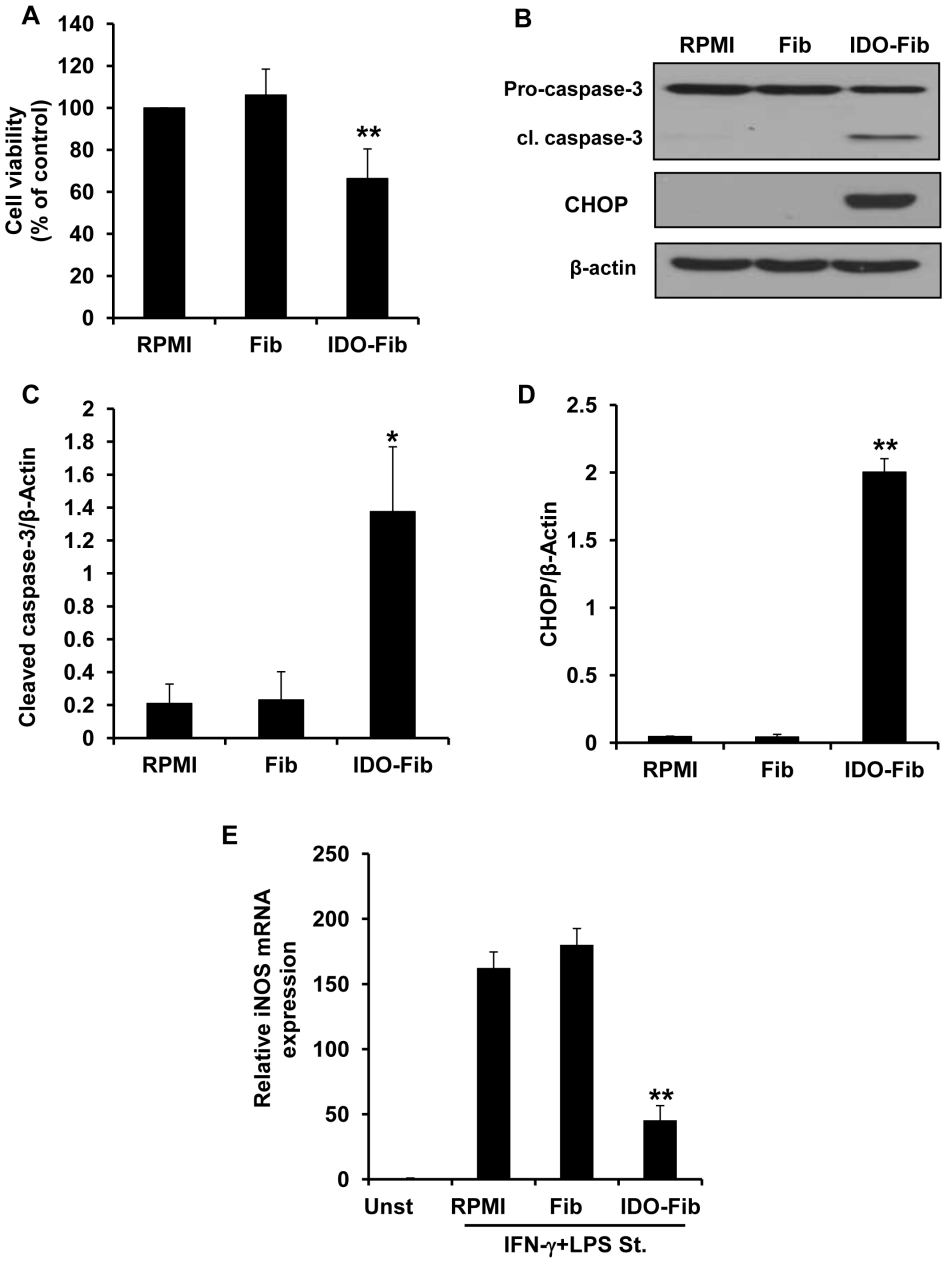
Effect of IDO expression by bystander fibroblasts on viability and proinflammatory activity of peritoneal macrophages. (A) Evaluation of the effect of IDO induced tryptophan deficiency/kynurenine accumulation on primary macrophage viability. Peritoneal macrophages were either cultured in RPMI or co-cultured with control (Fib) or IDO-expressing fibroblasts (IDO-Fib). MTT assay was done after 3 days of incubation. (B) Apoptosis induction in macrophages co-cultured with IDO-expressing fibroblasts. Presence of cleaved caspase-3 (cl. caspase-3) and CHOP was analyzed in macrophage cell lysate following 3 days of co-culture. The ratio of cleaved caspase-3/β-actin (C) and CHOP/β-actin (D) expression in macrophages, respectively. (E) Inhibition of proinflammatory activity in peritoneal macrophage co-cultured with IDO-expressing fibroblasts. iNOs expression was evaluated in INF-γ+LPS stimulated macrophages preincubated in PRMI or conditioned medium collected from either control (Fib) or IDO-expressing fibroblasts (IDO-Fib). GAPDH was used as the reference gene. Data is mean±SEM of three independent experiments (*P-value<0.05, **P-value<0.01, n = 3).

Primary macrophages isolated from mouse peritoneal space were co-cultured with normal or IDO expressing fibroblasts in order to evaluate the effect of IDO-induced tryptophan deficiency and kynurenine enrichment on the viability of these cells. As shown in figure 6A, the viability of macrophages co-cultured with IDO-expressing fibroblasts is markedly reduced (66.36±14.1%, ** P-value<0.01, n = 3) when compared with the cells cultured in RPMI or co-cultured with normal fibroblasts (106.13±12.33%). Presence of cleaved caspase-3 (an indicator of apoptosis) and CHOP (a GCN2 kinase pathway activation marker) were evaluated in the cell lysate of primary macrophages co-cultured with either normal fibroblasts or IDO-expressing fibroblasts. As shown in figure 6B, while no CHOP or cleaved caspase-3 expression was detectable in peritoneal macrophages cultured in RPMI or co-cultured with normal fibroblasts (lane Fib), a marked increase in the levels of cleaved-caspase-3 (cl. caspase-3) and CHOP was observed in macrophages co-cultured with IDO-expressing fibroblasts (lane IDO-Fib). Figures 6C and D, represent the quantitative analysis of cleaved caspase-3 and CHOP expression shown in figure 6B, respectively (* P-value<0.05, ** P-value<0.01, n = 3).

### IDO Induced Tryptophan Deficiency and High Levels of Kynurenine Inhibit iNOS Expression by Stimulated Primary Macrophages

In order to determine the effect of IDO induced tryptophan deprivation/kynurenine accumulation on the proinflammatory responses of macrophages, primary macrophages were treated with either normal or IDO-expressing fibroblasts conditioned medium for 1 hour and then stimulated with LPS+IFN-γ. Expression of iNOS was evaluated in these cells by using Q-PCR after 12 hours of incubation. As it is shown in figure 6E, while stimulated cells cultured in RPMI or normal fibroblast conditioned medium (Fib) express high levels of iNOS mRNA in response to LPS+IFN-γ, iNOS expression is significantly reduced (** P-value<0.01, n = 3) in peritoneal macrophages cultured in the IDO-expressing fibroblasts conditioned medium (IDO-Fib).

### IFN-γ Induces the IDO Expression in Primary Macrophages Cultured in Tryptophan Deficient Medium

To determine the effect of tryptophan deficiency on IFN-γ induced IDO expression, peritoneal macrophages were cultured in RPMI (R), Trp-D medium (T) or Trp-D medium supplemented with tryptophan (T+T) and stimulated with IFN-γ. As it is shown in Figure S2 treated macrophages cultured in tryptophan deficient medium (T) express high levels of IDO which is comparable to the cells cultured in RPMI or Trp-D supplemented with tryptophan (T+T) at the mRNA and protein level (Fig. S2A and C, respectively). Figure S2B and D represent the quantitative analysis of IDO expression shown in Figure S2A and C, respectively.

### IDO Expression Inhibits Macrophage and T-cell Infiltration into the Composite Grafts and Suppresses iNOS Expression by Macrophages

**Figure 7:**
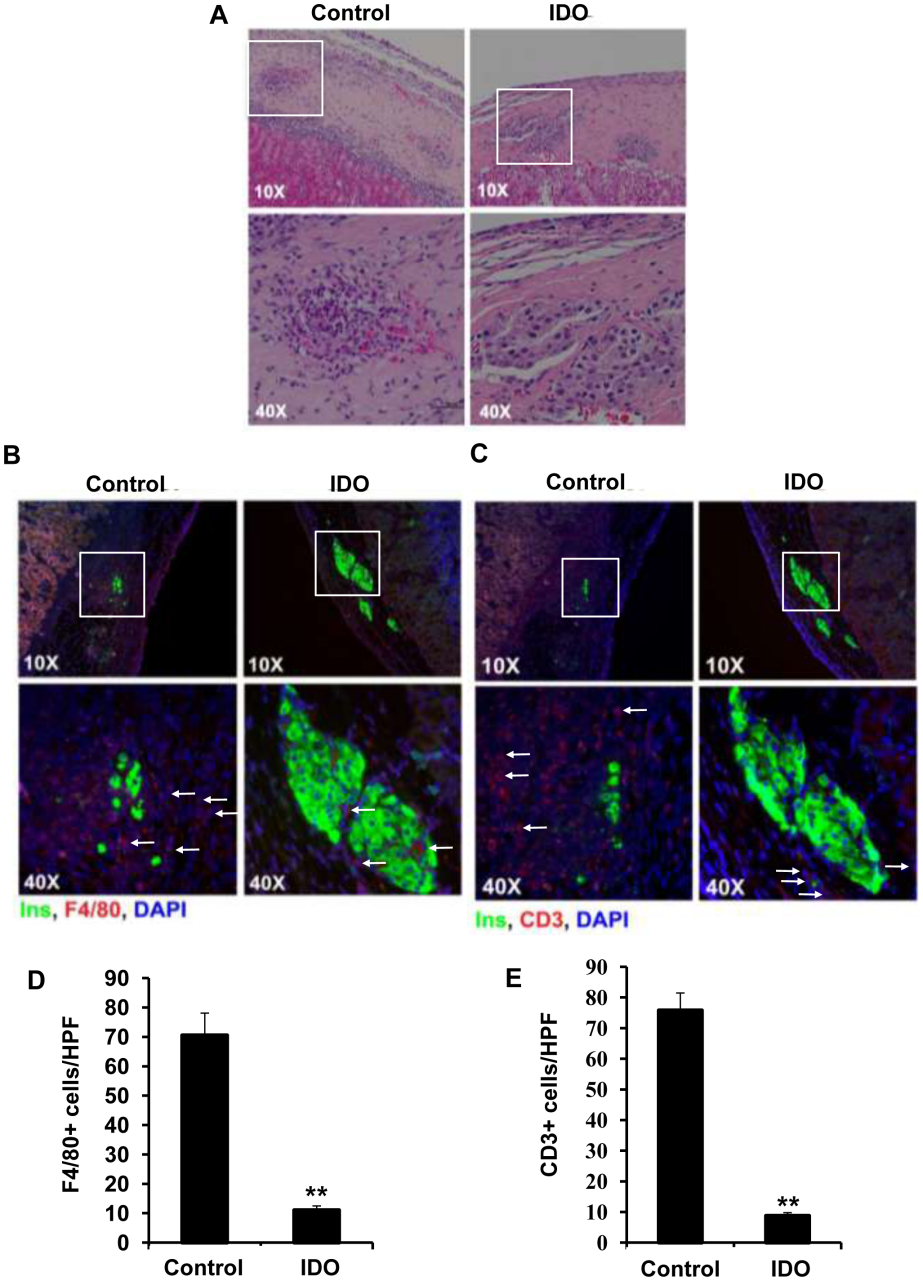
Infiltration of composite islet xenografts by F4/80^+^ and CD3^+^ cells. Three dimensional grafts were constructed by embedding isolated rat islets in the collagen matrix populated with IDO-expressing or control B6 mouse fibroblasts. Graft-recipient mice were killed 10 days after transplantation of the composite xenografts. Retrieved composite islet grafts were then subjected to H&E staining (A) and double immuno-fluorescence staining for F4/80 and insulin (B) or CD3 and insulin (C). The arrows indicate infiltrated immune cells. Quantitative analysis of F4/80^+^ macrophages (D) and CD3^+^ T cells (E) infiltration into islet xenotransplants. Graphs show manual counting scores±SEM per high-power field (HPF, x400). Data derived from the examination of four different HPFs per tissue section (**P-value<0.01, n = 4).

To investigate the immunosuppressive effect of local IDO expression on islet xenotransplantation, three-dimensional grafts were engineered by embedding 300 Long Evans rat islets within the collagen matrix populated with adenoviral-transduced IDO-expressing or control (untreated) B6 mouse fibroblasts. B6 recipient mice, transplanted with these composite grafts, were killed 10 days post transplantation to examine histopathological changes in islet xenografts. Recovered composite grafts were then stained with H-E or subjected to double immuno-fluorescence staining for either insulin and glucagon, F4/80 and insulin, CD3 and insulin or iNOS and F4/80. Histological studies demonstrated that islet morphology was well preserved in the IDO-expressing composite grafts; while control grafts were infiltrated by immune cells and islets were destroyed by 10 days after transplantation (Fig. 7A). Interestingly, in contrary to the a large number of cells (probably immune cells) which were present on the edge of xenogenic graft between the kidney tissue and the matrix composite, no cells were found in this area of the IDO expressing matrix graft.

To identify the types of infiltrating immune cells, a double staining of grafts for either F4/80 and insulin (Fig. 7B) or CD3 and insulin (Fig. 7C) was conducted and the result demonstrated a well preserved islet morphology in IDO expressing matrix composite whose beta cells express a very high level of insulin (Fig. 7B&C, IDO); while a very few beta cells in control composite remained undestroyed and expressed insulin (Fig. 7B&C, control). When these tissues were stained with either F4/80 or CD3+ as markers for infiltrating macrophage and T cells, the number of infiltrating immune cells was markedly lower in IDO expressing composites relative to those of controls. Minimal infiltration of macrophages was detected at the margin of IDO-expressing grafts. Statistical analysis of infiltrating T cells and macrophages into the islet composites revealed that IDO expressing xenografts demonstrated a significant reduction in the number of infiltrating F4/80^+^ macrophages (11±1.47 vs. 70.5±7.57 cells/HPF, IDO expressing graft vs. control, ** P-value<0.01, n = 4, Fig. 7D) and CD3^+^ T cells (8.75±1.03 vs. 75.75±5.72 cells/HPF, IDO expressing graft vs. control, ** P-value<0.01, n = 4, Fig. 7E) in comparison to control islet composites.

**Figure 8:**
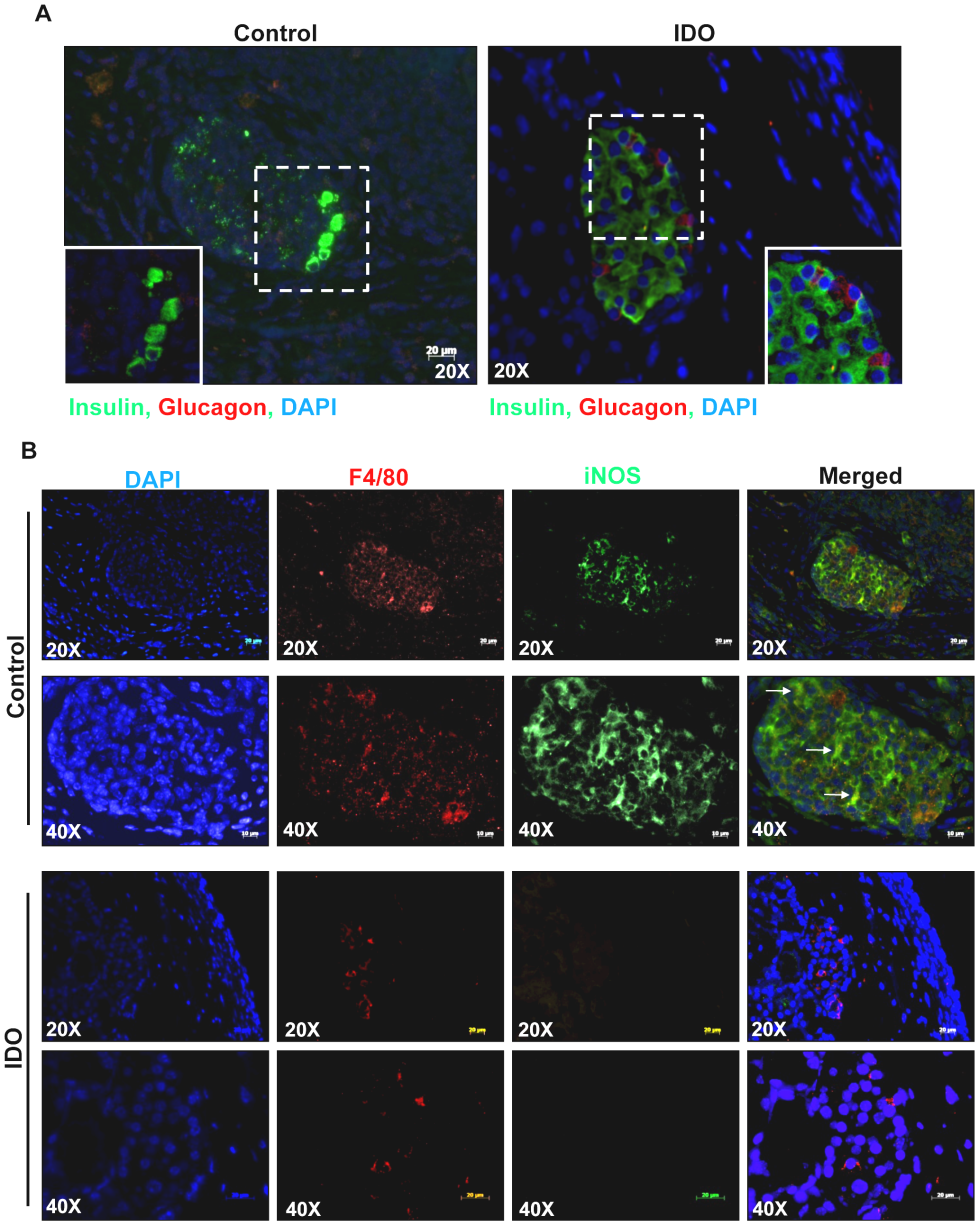
Local IDO expression impairs iNOS expression by infiltrating macrophages while keeping the islet functionality intact. Retrieved composite islet grafts were subjected to double immune-fluorescence staining for insulin and glucagon (A) and double immune-fluorescence staining for F4/80 and iNOS (B). The arrows indicate colocalization of iNOS and F4/80.

The islets in the IDO-expressing composite grafts were strongly stained for insulin and glucagon while the majority of the insulin and glucagon expressing cells were destroyed in the control graft (Fig. 8A). To evaluate the iNOS expression by infiltrating macrophages in composite islet grafts, a double immunostaining of grafts for F4/80 and iNOS was conducted; the result demonstrated high levels of iNOS expression, the signal of which is colocalized with F4/80 staining in control grafts. Minimal infiltration of F4/80^+^ macrophages was detected in the islets of the IDO-expressing grafts; however, iNOS was not detected in these cells (Fig. 8B).

## Discussion

A study by Miki *et al.* (2001) provided the first evidence on the immunoprotective effects of local IDO expression on the outcome of the allotransplantation. It was shown that administration of IDO inhibitor, 1-MT, in transplant recipients leads to loss of spontaneous tolerogenicity of liver allografts in mice [33]. The immunoprotective effects of IDO in allotransplantation have been reinforced by subsequent studies demonstrating the significant prolonged graft survival in response to overexpression of IDO in cornea [34], heart [35] and islet [8] transplantation. Li *et al*. (2006) and Wee *et al.* (2008) studies revealed that local IDO expression prolongs xenograft survival via inhibition of T cell infiltration into the graft and suppression of xenoreactive T cell proliferation [26], [36]. However none of these studies evaluated the effect of local IDO expression on innate immune responses involved in xenograft rejection. Regarding the pivotal role of innate immune responses, especially macrophages [37], in xenograft rejection, we sought to investigate the effect of IDO induced tryptophan starvation and kynurenine accumulation on macrophage viability, functionality and cell mediated islet xenograft rejection.

The specific immunosuppressive effects of IDO on adaptive immune cells have been studied extensively. It is now well accepted that IDO generates a tryptophan-deficient and kynurenine-rich microenvironment that can cause selective induction of apoptosis in CD4^+^/CD8^+^ T cells and human PBMCs [38]. In this study we demonstrated that peritoneal macrophage co-culture with IDO expressing fibroblasts significantly reduces the viability of macrophages via apoptosis induction in these cells. We also showed that the tryptophan deficiency, rather than high levels of kynurenine, significantly decreases Raw264.7 cell viability through activation of pro-caspase-3. Activated cleaved caspase-3 subsequently cleaves and deactivates PARP, involved in DNA damage repair, and initiates the programmed cell death. Pro-apoptotic effect of tryptophan starvation on Raw264.7 cells was verified by inhibition of apoptosis in response to addition of tryptophan to the tryptophan-deficient medium. It was previously shown that the specific pro-apoptotic effect of IDO induced tryptophan deprivation in immune cells is mediated by selective activation of GCN2 kinase pathway in these cells in comparison to non-immune cells such as fibroblast or islets [22], [23], [39]. As it is shown here, Raw264.7 cells cultured in Trp-D medium and primary macrophages co-cultured with IDO expressing fibroblasts demonstrated a significant up-regulation of CHOP, a downstream signaling molecule in GCN2 kinase, in comparison to cells cultured in RPMI, Trp-D+Trp medium or co-cultured with control fibroblasts, respectively. This data suggest that while maintaining the normal functionality of islets, IDO induced tryptophan deprivation reduces macrophage viability by inducing apoptosis in these cells.

A study by Yi *et al*. in 2003 provided direct evidence about the ability of T cell-activated macrophages in specific recognition and rejection of islet xenograft [7]. These activated macrophages produce excessive amounts of NO which has cytotoxic and cytostatic effects and causes direct tissue damage [40]. Several studies provided indirect and direct evidence for the involvement of NO in graft rejection. A significant increase in the iNOS expression and NO production has been reported in acute graft rejection [41], [42]. Vos *et al.* study (2000) also revealed that administration of iNOS blockers after transplantation improves renal allograft function and reduces graft injury [43]. In this study we demonstrated that peritoneal macrophages, treated with the IDO-expressing fibroblast conditioned medium, shows significantly lower levels of iNOS expression in response to LPS+IFN-γ stimulation. We also showed that tryptophan deficiency rather than kynurenine enrichment inhibits iNOS expression and NO production by stimulated Raw264.7 cells. The specific inhibitory effect of tryptophan deficiency on iNOS expression and NO production by activated macrophages was further confirmed by the restored macrophage proinflammatory activity in response to tryptophan supplementation to the Trp-D medium. In 1997 Sekkai *et al*. demonstrated the inhibitory effect of 3-hydroxyanthranilic acid, a kynurenine pathway metabolite, on iNOS expression and enzymatic activity via inhibition of NF-κB activation [44]. They also showed that other tryptophan metabolites including kynurenine have no significant inhibitory effect on NF-κB activation [44] which support our data related to the non-inhibitory effect of kynurenine on iNOS expression and NO production. This study provides evidence for inhibitory effects of tryptophan deprivation on macrophages proliferation, survival and proinflammatory activity.

In order to evaluate the immunoprotective effects of local IDO expression, xenografts, rat islets imbedded in an IDO expressing fibroblast populated matrix, were transplanted in B6 mice. Using IDO expressing fibroblast we were able to demonstrate that local IDO expression inhibits the infiltration of CD3^+^ T cells into the xenogeneic islet graft. This is in agreement with other studies using stable IDO expressing cell line or IDO expressing bystander fibroblasts [8], [36]. Additionally as it is shown in this study, local IDO expression by bystander fibroblasts significantly inhibits the infiltration of macrophages into the islet xenografts and impairs their proinflammatory responses. Keeping in mind that kidney capsule is a confined micro-environment, the high levels of IDO expression by fibroblast-populated islet composite leads to a remarkable reduction in the tryptophan concentration and kynurenines accumulation *in vivo* at the graft site. While not negatively affecting the islet function, this low-tryptophan microenvironment protect islet xenografts by inhibiting the infiltration, proliferation and inflammatory activity of immune cells.

In conclusion this study is the first to demonstrate modulation of the innate immune response by IDO as a result of creating a tryptophan deficient environment. Specifically we have shown that IDO immunomodulatory effects might be mediated via inhibition of proliferation, induction of apoptosis or impairment of proinflammatory responses in macrophages. Our *In vivo* study demonstrated that macrophage and T cell infiltration into a xenogenic islet graft is significantly reduced in IDO expressing grafts and iNOS expression is inhibited in these infiltrating macrophages. Data generated from this study along with those previously reported by our group [8], [24], [25] support the feasibility and applicability of using IDO expression as a local immunosuppressive strategy for protecting both allo- and xeno islet grafts from immune rejection.

## Supporting Information

Figure S1 Adenoviral vector mediated IDO expression in transfected B6 fibroblasts. (A) Mouse dermal fibroblasts were infected with an adenoviral vector carrying human IDO cDNA. IDO expression was analyzed by Western blotting. (B) IDO/β-actin expression ratio was calculated in dermal fibroblasts. (C) kynurenine assay. IDO functional activity was determined by measuring the content of kynurenine in the fibroblast conditioned medium. Data is mean±SEM of three independent experiments (**P-value<0.01, n = 3). (TIF)

Figure S2 IDO expression by primary macrophages in response to IFN-γ stimulation. Primary macrophages, cultured in either RPMI (R), tryptophan deficient medium (T) or tryptophan deficient medium supplemented with 50 µg/ml of tryptophan (T+T), were stimulated with IFN-γ (1000 U/ml). (A) IDO mRNA expression in the treated macrophages. (B) IDO mRNA/β-actin expression ratio. (C) IDO protein expression in the IFN-γ treated macrophages. (D) IDO/β-actin expression ratio. Data is mean±SEM of three independent experiments. (TIF)
