# Supplementary figures and images for: Immuno-Regulatory Function of Indoleamine 2,3 Dioxygenase through Modulation of Innate Immune Responses
Source: PLoS One. 2013 Aug 5;8(8):e71044. doi: 10.1371/journal.pone.0071044 (PMC3733714; doi:10.1371/journal.pone.0071044)

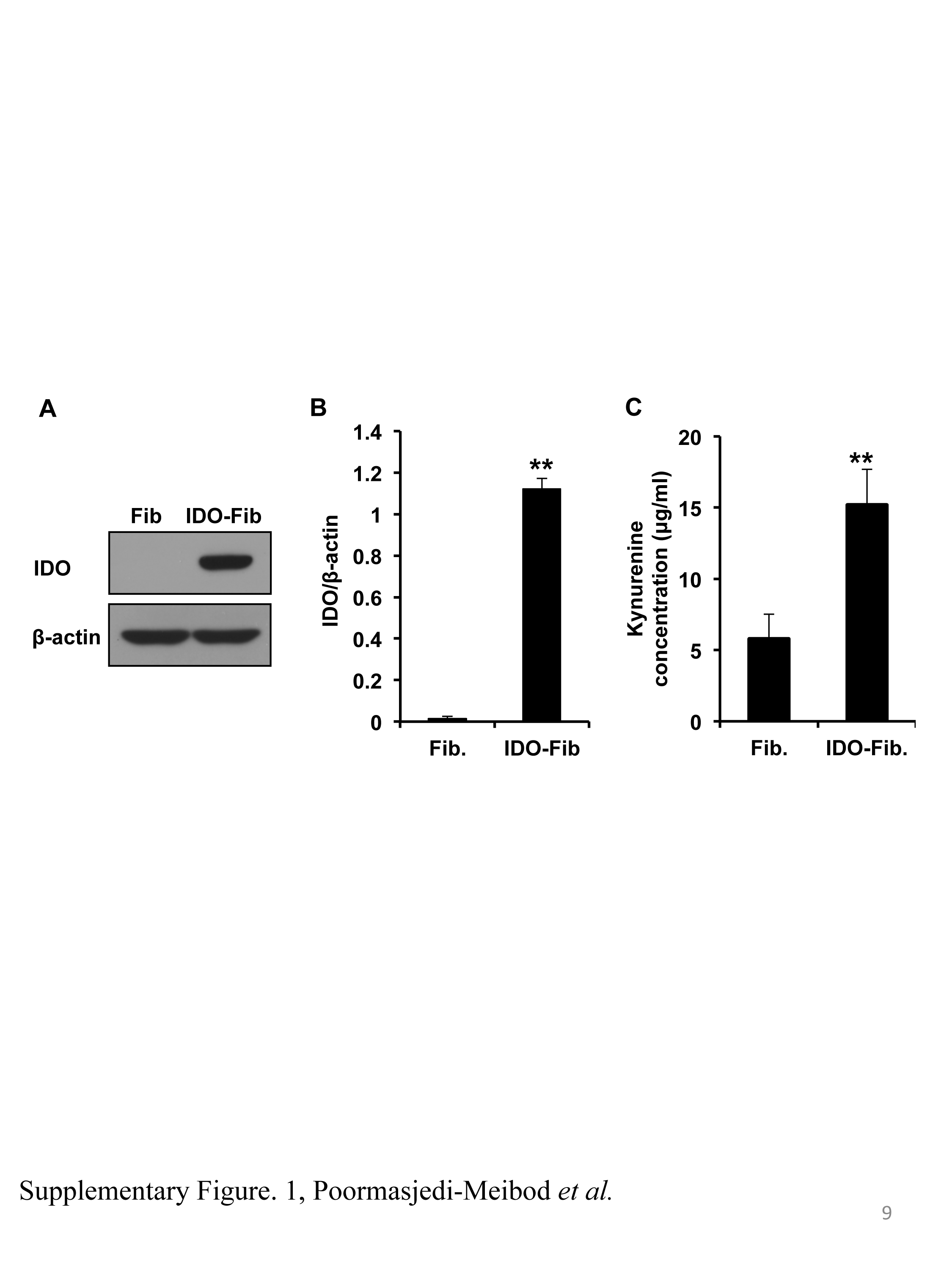

Supplement: Figure S1 — (TIF) [file pone.0071044.s001.tif]

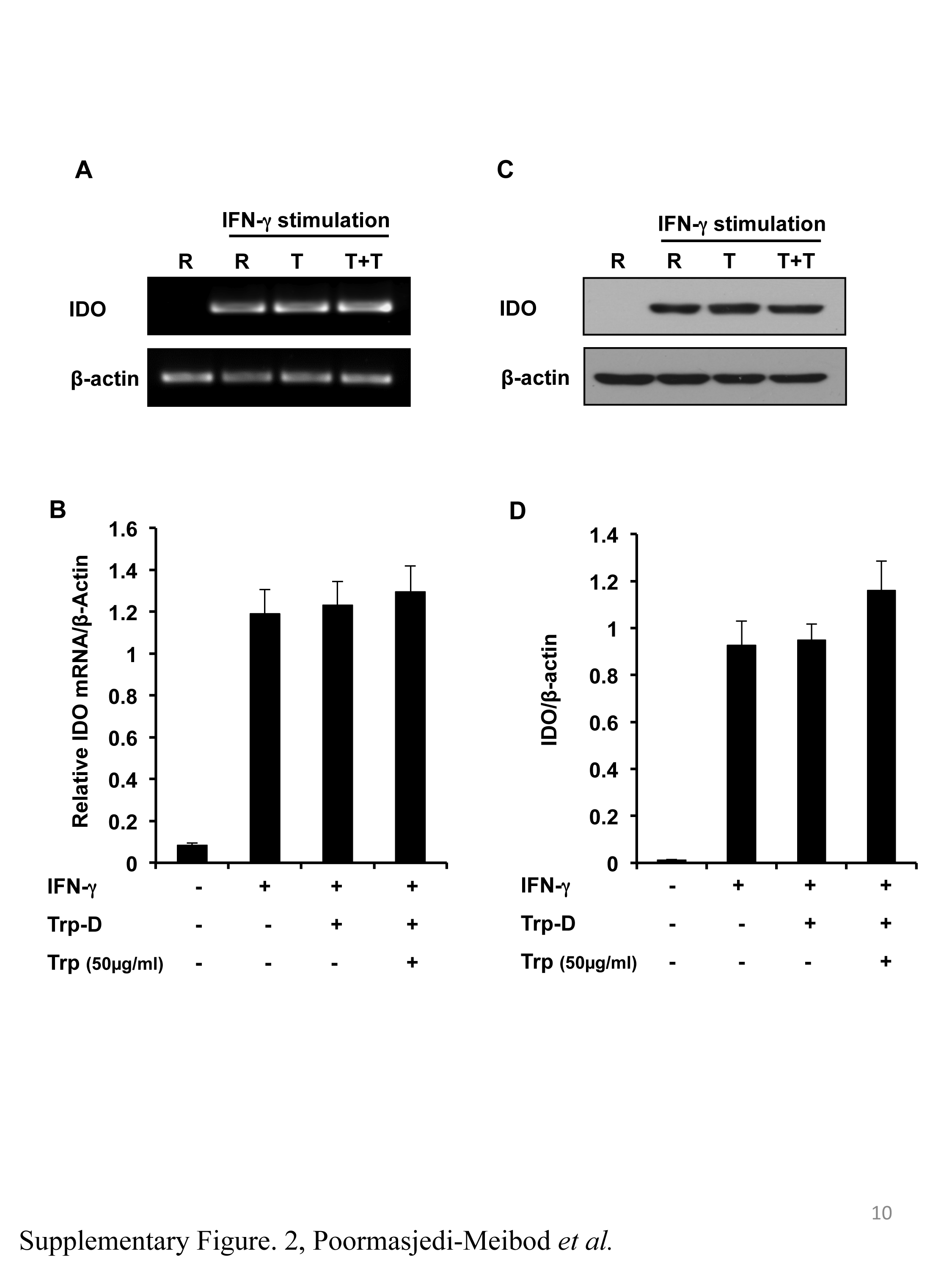

Supplement: Figure S2 — (TIF) [file pone.0071044.s002.tif]
